# Understanding physical climate risks and their implication for community adaptation in the borana zone of southern Ethiopia using mixed-methods research

**DOI:** 10.1038/s41598-023-34005-1

**Published:** 2023-04-27

**Authors:** Mulugeta Shibru, Alfred Opere, Philip Omondi, Maina Gichaba

**Affiliations:** 1 Department of Earth and Climate Sciences, Faculty of Science and Technology, University of Nairobi, Nairobi, Kenya; 2 IGAD, Climate Prediction and Applications Centre (ICPAC), Nairobi, Kenya

**Keywords:** Climate change, Natural hazards

## Abstract

Pastoralists in the Borana zone of southern Ethiopia are grappling with climatic risks and impacts, and their livelihoods appear to be precarious. The long-term changes in precipitation, temperature and extreme events are under-researched aspects of climate risk in the study area. Climate observations, local people’s experiences, and memories of weather and drought patterns were analysed in this work to better understand the climate risks in the study region and provide actionable knowledge to facilitate adaptations. In southern Ethiopia, the primary drivers of climate risks are rainfall variabilities, rising temperatures, and drought. The annual rainfall variability observed in the study area (20–35%) is greater than the overall estimate for Eastern Africa (15–25%), indicative of a heightened climate risk in this area. Furthermore, seasonal and intra-seasonal rainfall variabilities are being aggravated by rising temperatures, leading to increased frequency and magnitude of extreme events, particularly in the lowland area. These changes, specifically droughts of different intensities occurring every 2–3 years, erode the livelihood of the pastoralists. The lack of consensus among stakeholders as to the causes and aggravating factors of these climate changes impedes adaptation actions. The authors recommend the importance of initiating a participatory platform that will facilitate discussion on climate change to create a common understanding of the problem and relate it to public policy. The use of gridded precipitation and temperature data is recommended in future research to improve the availability of climate information for climate risk management. In addition, the use of mixed methods and local knowledge, as demonstrated in this study, is highly recommended to better understand climate risks, particularly when there is a limited availability and quality of long-time-series climate data.

## Introduction

Climate change is one of the greatest challenges of the twenty-first century in nearly every country of the world, regardless of the level of development. However, underdeveloped countries are more affected by the impacts of weather-related losses than industrialized countries^1^. Drylands currently cover approximately 46% of the global land area and are home to 3 billion people, 90% of whom live in developing countries. Climate change is presenting an unprecedented threat to drylands with far-reaching environmental, social, and economic consequences^2^.

A significant area of these drylands is climatically classified as Arid or Semi-Arid Lands (ASALs), meaning that rainfall has a patchy distribution in both time and space. Drylands additionally receive relatively low overall amounts of precipitation in the form of rainfall or snow^3,4^ and are characterized by a high degree of inter-annual rainfall variability. Extreme climatic and weather events are inherent climatic characteristics in the ASALs of Africa^5^. The extreme climate and weather condition is mainly attributed to La Niña, a natural large-scale cooling of ocean surface temperature in the central and eastern equatorial Pacific Ocean. For example, heavy rainfall events, floods, land-falling tropical cyclones, droughts, heatwaves, wildfires, and sandstorms affected the African continent in 2021^6^. These events affected diverse climate-sensitive sectors in Africa, impacting agriculture, food security, water resources, population displacement, health, and transportation^6^.

Dryland ecosystems are fragile and highly sensitive to interactions between human activities and climate change^7^. The heavy reliance of dryland populations in developing countries on climate-sensitive natural resources for their livelihoods and their limited adaptive capacities to climate change contribute to their vulnerability status^8,9^. These ecosystems have equally undergone substantial anthropogenic changes via urbanization and land-use changes^7^, and these changes have accentuated the degrees of climate variability, change, and fragility of the ecosystems.

The ASALs of East Africa, including the study area, are not exceptional to the pattern of climate change and its impacts observed in global drylands. In East Africa, rainfall variability is the main climate and weather element affecting the ecosystem services, agricultural production, and socioeconomic development of the region and is exacerbated by frequent drought and flood events^10–12^. The successive drought episodes that hit many parts of the Horn of Africa since the 1990s (1990s, 2000–2001, 2005–2006, 2008–2009, 2010–2011, and 2015–2016) were evidence of increasingly high risks associated with substantial and seemingly increasing climatic variabilities^13–18^. The devastating drought observed in the East African countries of Ethiopia, Kenya, and Somalia during 2020–2022 is attributed to prolonged La Nina weather events in addition to climate variabilities. The Intergovernmental Panel on Climate Change (IPCC) Special Report on Extremes^19^ established a linkage between climate risk, particularly extreme events, and natural disasters. The Borana pastoralists in southern Ethiopia, like others in the region, are dependent on climate-sensitive natural resources for their livelihoods. Rainfall and temperature are the two most important variables that affect these key resources, and their variabilities contribute to extreme events such as drought and flood and exert significant impacts on the livelihoods of the population, socioeconomic development, and local ecosystems. However, climate variability is not entirely a new phenomenon in the Borana pastoral system. The traditional management strategies of people living in this region are adaptive, with embedded social structures and resource management systems that respond to varied climates^20^. Pastoralists are the custodians of the most productive indigenous cattle breeds of East Africa, which are recommended as suitable for ASALs^21^, where climate variability and harsh environmental conditions are common features. However, the indications are that the prevailing climate variabilities are becoming extreme, and the management strategies of pastoralists are being overstretched. The four consecutive failed seasons recently experienced in southern Ethiopia like East African countries serve as evidence of increasingly extreme events and devastating impacts beyond the coping capacities of the pastoralists.

Borana pastoralists suffered three major droughts between 1980 and 2000 in which they lost approximately 35–67% of their livestock worth hundreds of millions of USD^22,23^. Between 2005 and 2016/2017, pastoralists experienced at least four major droughts that contributed to progressive losses of livestock assets^24–28^. Most previous studies have focused on drought as a hazard and analysed its impacts and the ways pastoralists cope with those effects based on ex-post-facto studies. The long-term changes in precipitation and temperature are relatively under-researched aspects of climate risk that affect the current and future vulnerability of the studied ecosystem. Moreover, the engagement of pastoralists in the research process has been limited despite the importance of local knowledge in understanding climate risks and related hazards, especially in contexts where weather and climate monitoring systems are weak. This insufficiency in past research has affected the common understanding of the problem and the utility of the research outcomes in informing climate change policies and community adaptation strategies.

The frequency, intensity, and cost of natural hazards are expected to further increase in the coming decades (1). This predicted trend calls for understanding and addressing climate risks at local and global scales to mitigate growing disasters. This research is a response to such a call. The goal of this work is to understand the climate risks faced by pastoralists in the Borana zone of southern Ethiopia and provide actionable knowledge and information to facilitate their adaptation. This research addresses the observed research gap specifically by (a) characterizing the historical patterns of climate variability, climate change and drought using climate observations, (b) integrating climate observations and local knowledge to provide a contextual understanding of climate risks, and (c) identifying policies and adaptation support that pastoralists may need to withstand climate risks and their impacts.

## Methods

In this section, the area of study is described, followed by the data collection methods and analysis used to achieve the objectives of the research.

### Study area

**Figure 1:**
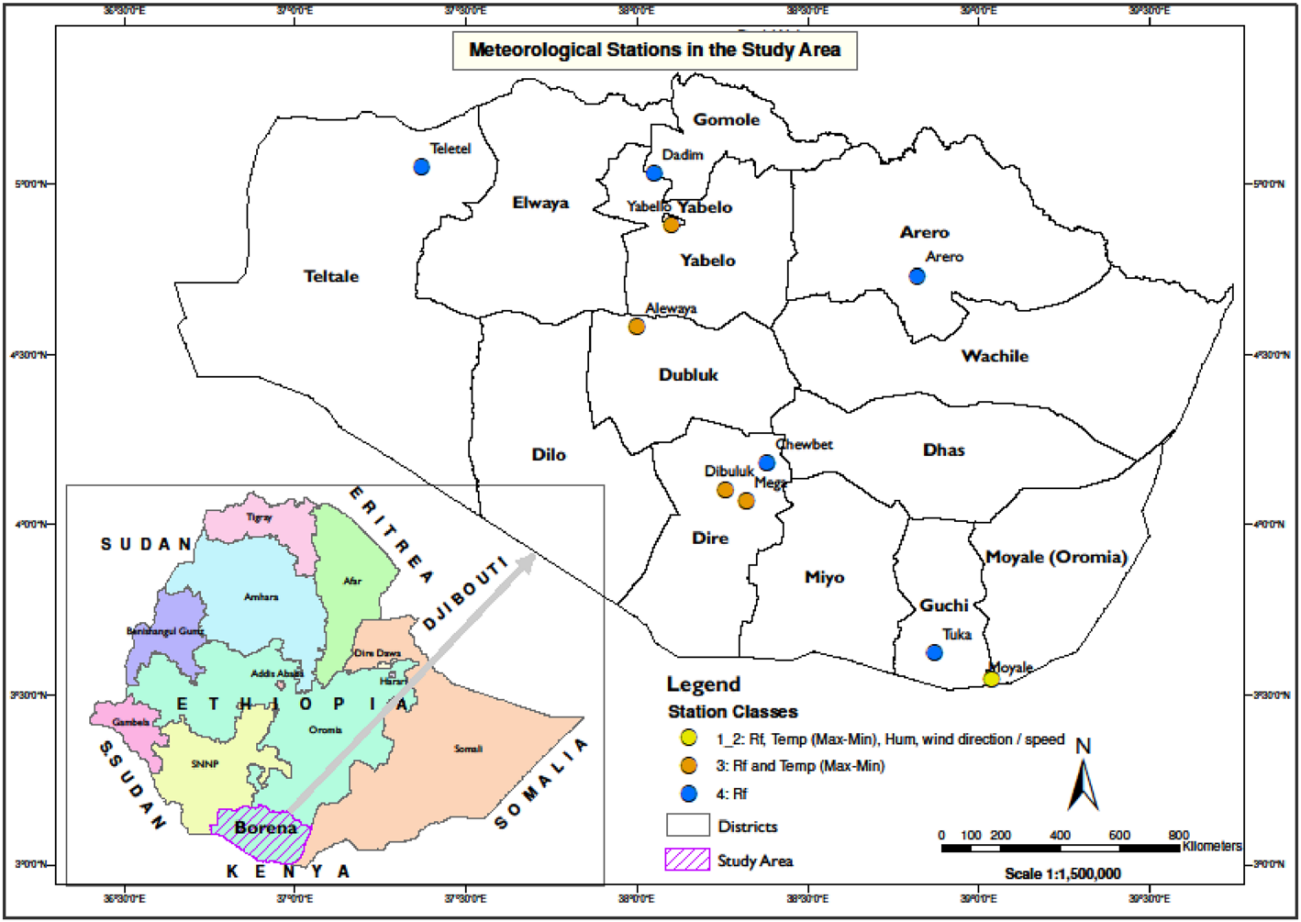
Study area and locations of meteorological stations. (Source: Humanitarian Data Exchange-Ethiopia Shape file and NMA station GPS points).

This study was conducted in the Borana zone, one of the twenty administrative zones of the Oromia Regional State of Ethiopia. This zone occupies an area of approximately 55,711 km^2^ (20% of the regional surface area), located between 3°36 and 6° 38′ North latitude and between 3°43′ and 39° 30′ East longitude and bordering Northern Kenya. The zone is subdivided into thirteen districts, as shown in Fig. 1.

The rainfall in the study area follows a bimodal climatology with the main rainfall period spanning from March to May, locally called “Genna”, and short rains occurring from September to November, called “Hagaya”. Each of the rainy seasons is followed by a dry season, i.e., the cold dry season, *“Adolessa”* (June–Aug), and the long dry season, *“Bonna Hagaya”* (Dec–Feb). The area receives average annual rainfall between 286 and 896 mm, with considerable inter-annual variability ranging from 18 to 69%^25^.

The zone is one of the warmest lowland regions of Ethiopia, with an annual mean temperature ranging from 20 to 25 °C^29^. March to May is considered the warmest period, while November to January is considered the coldest period^30^.

### Data collection methods

#### Climate data collection

Meteorological data are one of the main data sources collected to address the objectives of this study. The National Meteorological Agency (NMA) of Ethiopia has established a number of weather stations across the country, including within the study area, to collect observational data regularly. Most of the stations in the Borana zone were established in the late 1980s, with the exception of the Yabello and Mega stations. Eleven stations are operational. NMA classifies these stations based on the weather data collected. Stations that record precipitation data are classified as class 4, while those that record both precipitation and temperature data are considered class 3. Class 1–2 s station records relative humidity and wind direction/speed data in addition to precipitation and temperature data. Most of the stations in the study area are considered class 4 or 3. There is only one Class 1–2 station included. All available station-recorded daily data used in this study, spanning from 1986 to 2018, were collected from the NMA. Figure 1 summarizes the the data availability for each station*.*

#### Remotely sensed data

Moderate-Resolution Imaging Spectro-radiometer (eMODIS) normalized difference vegetation index (NDVI) data processed by the United States Geological Survey (USGS) were widely used by the Famine Early Warning Systems Network (FEWSNET) for drought monitoring. The Dekadal (10 days) raster datasets from 2002 to 2020 were acquired from the FEWSNET data portal to ascertain trends in NDVI and complement the observed data.

#### Household data

Household demographic characteristics, common livelihood risks in order of importance, and perceptions of drought, climate variability, and change trend, among other questions, were included in the closed-ended survey instrument. Rainfall (amount, rainy days, timing- onset or cessation, and distribution), temperature, drought (frequency, duration, severity, and intensity), and flood (frequency) were key parameters included in the instrument. The past 10 years were used as the “recall period” for the trends of the perception questions given the age variation of the households. The instrument was digitized into Open Data Kit (ODK) tools and facilitated mobile data collection using a smartphone. The detailed sampling procedure, sample size, and sample distribution in the study area are presented in Annex 1.

#### Qualitative data

Focus group discussions (FGDs) and key informant interviews (KIIs) were the qualitative assessment methods applied in this study to complement the climate and household data. A mixed focus group interview was facilitated due to the extensive nature of the study area. Women, men, youth, elders, and various ethnic group representatives constituted the FGD. Community sketch/resources map, common livelihood risks ranked in order of importance, and perceptions of drought, climate variability, and change trends, among other factors, were included among the semi structured questions. The past 30 years were used as the reference period for the perception questions. Twelve FGDs were facilitated in total, one per district, with the exception of Teletel (two FGDs) because of its size and diversity of livelihoods (refer to Annex 2 for a map of the FGD distribution).

Diverse groups endowed with first-hand knowledge of the subject matter were used as key informants. Traditional grazing managers locally called “Jarsa Deheda” were one of the categories of KIs. The research team interviewed four of the five recognized grazing managers in Borana, i.e., Globo, Malebee, Wayama, and Gomole. The Dire grazing manager was not available due to his medical condition. Some of the indigenous weather forecast practitioners were also used as key informants. One of the known animal behaviourists *(“Waragu”)* and cyclical calendar practitioners in Borana *(“Mara”)* were interviewed as KIs. Discussions with representatives of relevant government departments of the Borana zone were further used as KIIs. A total of seven KIIs were facilitated by the research team.

Semistructured questions similar to the FGDs were included to ascertain the key informants' understanding. Drought (frequency, duration, severity, and intensity), flood (frequency), rainfall (amount, rainy days, timing- onset or cessation, and distribution) and temperature were key parameters included in the instrument. Likewise to the focus group discussions, the past 30 years were used as the reference period for the perception questions. The “timeline” of major drought events was expanded to 60 years by most of the KIs during the interview process.

#### Secondary data sources

The United Nations Office for Disaster Risk Reduction (UNDRR) has collated disaster-related losses and damages that prevailed in Ethiopia from 1994 to 2013 from various sources. These digitized data are available on the Humanitarian Data Exchange (HDX) platform. Major historical disaster data relevant to the study were downloaded from HDX. These data were used to complement the historical timeline of the key informants to characterize prevailing disasters in the study.

### Quality control of meteorological data

**Figure 2:**
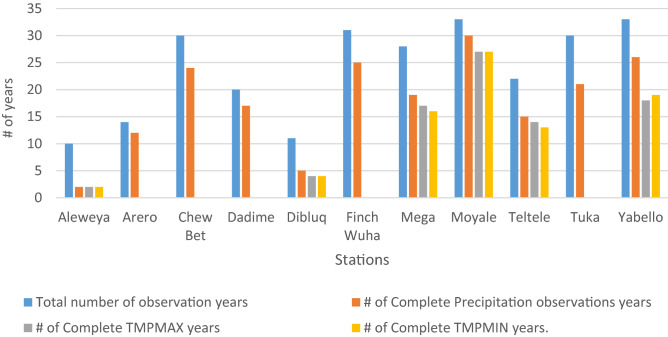
Number of years with complete monthly rainfall/temperature observations compared to the total years of observation by station.

The acquired climate data were further assessed for completeness and quality before analysis. All of the stations suffered from data gaps and incomplete historical records, with some lapses in reporting being larger than others. Figure 2 shows the total number of years that each station has reported data relative to the number years of complete observations (no missing data). The annual observations of a given station were considered “complete” when rainfall or temperature data were available for 10 months or more in a given year.

Climate data often have missing information, which limits their use^31^. Stations with significant years of incomplete data are believed to affect the overall statistical results obtained using the time-series data. Estimating missing data is a routine procedure to address these gaps, and there are standard methods of doing so. The arithmetic mean method, normal ratio method, modified normal ratio method, and interpolation method are some of the most widely practised methods. Missing rainfall can be determined using a simple arithmetic average if the normal annual precipitation at various stations is within 10% of the normal precipitation^32^. If the normal precipitation varies considerably (exceeding 10%), then missing rainfall can be estimated by weighting the precipitation at various stations by the ratios of the normal annual precipitation^33^. The other method used to fill missing meteorological variables is the use of datasets from other selected stations in the surrounding area and the application of appropriate spatial interpolation methods^34^. Most of these methods, however, require the availability of well-established climatological normal information of base stations as well as neighbouring stations with grossly missing data in the study area.

In addition, a considerable estimation level is required to fill in the prevailing station data gaps that affect parameter estimations. This study used different approaches to address the data gaps in the absence of effective estimations. First, we prioritized stations with a reasonably complete data. For precipitation, these stations were Chewe Bet, Finchwoha, Yabello, and Moyale. For temperature, only one station has acceptable data coverage for prioritization: Moyale. Second, we decided on a common window period for the analysis of prioritized stations. The 1990–2017 period had a lesser degree of missing values and thus a smaller estimation requirement than other periods across the stations. Hence, this period was chosen as the common window for the analysis of prioritized stations. the missing monthly rainfall data were filled using the corresponding monthly median of each station. The median was selected as it is a better representation of the central tendency, relative to the arithmetic mean, given the prevailing extreme values of rainfall data in the study area.

**Figure 3:**
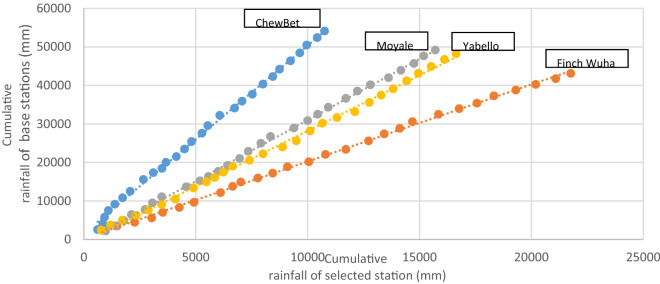
Double mass curves of the selected rainfall stations in the study area.

The double mass curve (DMC) is the most commonly used graphical approach used to test the homogeneity of time-series data at prioritized stations, as presented in Fig. 3. Its low data requirement makes this technique popular, and the method can indicate a change in the slope of data as a breakpoint from which the data exhibit inconsistency. The prioritized stations were found to be consistent according to the double mass curve analysis. Some of the minor breakpoints observed at some of the stations along the trajectory emanated from the data estimations or from changes in the observing conditions, practices, or procedures over time.

### Data analysis methods

#### Climate data analysis

Descriptive statistics are a powerful tool for summarizing different climatic parameters within specific time periods. The mean and median are a useful measures of the central value of observed elements such as precipitation and temperature and is used to approximate normality. The standard deviation (SD), standard error (SE), and coefficient of variation (CV) are among the selected dispersion statistics used to depict the extent of climate variability compared to the normal state. The selected statistics were calculated for a series of different observational periods (i.e., the monthly, seasonal and annual periods).

Parametric trend analysis (statistical or graphical) methods are used to ascertain trends in observed and remotely sensed time-series data. The nonparametric Mann–Kendall (M–K) test and Sen’s slope estimator were analysed herein using XLSTAT 2017 software to determine the direction, statistical significance, and magnitude of the trends of the observed and remotely sensed data.

The rainfall anomaly index (RAI**) **was used to characterize the severity of drought stress in the study area. The RAI is a simple drought index used to analyse the frequency and intensity of dry and wet seasons or years and was developed by van Rooy in 1965^32^. It incorporates a ranking procedure to assign magnitudes to positive and negative precipitation anomalies. The equation of the index is shown as follows:

Seasonal/annual rainfall anomaly index (RAI):

1 $$RAI=\pm 3\frac{N-\overline{N}}{\overline{M}(\overline{X)}-\overline{N}}$$

where: $N$: current seasonal/yearly rainfall for the RAI generation period, $\overline{N}$: seasonal/yearly average rainfall in the historical series (mm), $\overline{M}$: average of the ten highest seasonal/yearly precipitation values in the historical series (mm), $\overline{X}$: average of the ten lowest seasonal/yearly precipitation values in the historical series (mm).

RAI analysis can further help researchers determine the probability of occurrence of dry conditions, including the drought and recurrence intervals, in the study area. The probability of drought occurrence by severity was calculated using the equation below.

Probability of occurrence of dry conditions:

2 $$P=\frac{n}{N}$$

where: P: absolute probability of occurrence (severity), n: number of event occurrences (RAI < − 0.99) of drought, N: Total number of possible occurrences (the study period).

The dry and drought condition recurrence intervals or return periods for each of the stations were computed from the RAI based on the formula below:

Recurrence interval or return period:

3 $$Ri=\frac{Time \left(Years\right)}{Number \; of \; events (RAI \; less \; than -0.49 \; occurred)}$$

#### Household and qualitative data analysis

The frequency distribution and simple graphic analysis were used to analyse the household data using a statistical package for the social sciences (SPSS) and Microsoft Excel. Each of the focus group discussions and key informant interview audio records was transcribed as the first step of the qualitative analysis. The transcriptions were further edited, synthesized, and analysed using the content analysis technique. The mapping, ranking, timelines, trend analysis, and ratings collated from the participatory tools provided perspectives for the subsequent qualitative data analysis.

## Results

### Rainfall and temperature descriptive analyses

The mean, median and SE statistics for annual and seasonal precipitation were summarized for the selected stations and are presented in Table 1. The contribution of each rainy season to the annual rainfall total was also calculated and is provided in Table. In addition, the annual and seasonal average temperatures for the Moyale station are presented in Table 1.

**Table 1:** Summary statistics of temperature, precipitation and contribution of each season to annual rainfall (1990–2017). Source (Author): Computed based on data from the NMA of Ethiopia.

Station	Annual mean ± SE and (median) in mm	Seasonal mean ± SE and (median) in mm		Contribution of each season to the annual rainfall in %	
		Genna	Hagay	Genna	Hagay
Chew Bet	384.2 ± 25.7 (380.5)	196.2 ± 16.9 (185.1)^a^	143.5 ± 18.1 (136.9)	52	36
Finch Wuha	776.5 ± 36.7 (757.3)	375.8 ± 24.2 (351.8)	255 ± 28.5 (234.7)	49	32
Moyale	561 ± 38.8 (516)	258.9 ± 21.5 (242.6)	197.1 ± 29.5 (170.8)	47	35
Yabello	594.2 ± 24.3 (530.9)	296.8 ± 16.2 (301.7)	196.4 ± 17.4 (185.9)	50	32

Moyale	Average ± SE (°C)	Seasonal Mean ± SE (°C)			
		Genna	Adolessa	Hagay	B.Hagaya
MAX TMP	27.5 ± 0.12	28 ± 0.2	24.8 ± 0.1	26.7 ± 0.1	30.5 ± 0.3
MIN TMP	17.7 ± 0.2	18.5 ± 0.2	16.3 ± 0.2	17.3 ± 0.2	18.6 ± 0.3

Long rainy season, “Genna” (Mar–May); short rainy season, “Hagaya” (Sep–Nov); cold dry season, “Adolessa” (June–Aug) and long dry season, “Bonna Hagaya” (Dec–Feb).

^a^Median.

The coefficient of variation was calculated and presented in Table 2 to show the year-to-year or season-to-season rainfall variability. These statistics consider the deviations from the averages regardless of whether a station experienced high or low rainfall and can be used for relative comparisons of variabilities among stations. According to a cited work^35^, the CV can be used to classify the degree of variability of rainfall events as less (CV < 20), moderate (20 < CV < 30), high (CV > 30), very high (CV > 40%) and extremely high (CV > 70%) interannual rainfall variability.

**Table 2:** Annual and seasonal coefficients of variation (CV) in % for selected stations. Source (Author): Computed based on data from the NMA of Ethiopia.

Name of station	Coefficient of variation (CV) in %		
	Annual precipitation	Genna precipitation	Hagay precipitation
Chew Bet	35	46	67
Finch Wuha	25	38	60
Moyale	37	44	79
Yabello	21	34	49

### Rainfall and temperature trend analyses

#### Parametric trend analysis

The total annual and seasonal rainfall totals of a given station are plotted in a line graph over time to ascertain any observable trends. A similar analysis was performed for the Moyale station mean temperatures. A simple linear regression equation was generated using MS Excel to predict the annual precipitation in mm or mean temperature in $\mathrm{^\circ{\rm C} }$ for a unit change of the dependent variable (time in a year). In addition, linear trend lines and R-squared statistics were generated from the built-in tool to determine the direction of change and accuracy of the fit. The slope of the equation (trend) and R-square value for each station were extracted from line graphs and are presented in Table 3.

**Table 3:** Seasonal and annual precipitation/temperature slopes of the linear regression and R^2^ values. Source (Author): Computed based on data from the NMA of Ethiopia.

Station	Direction of trend line and associated R^2^				
	Annual precipitation	Genna precipitation	Hagay precipitation		
Chew Bet	+ 4.0784^a^ (0.0608)^b^	+ 2.8578 (0.069)	+ 1.6181 (0.0194)		
Finch Wuha	+ 4.573 (0.0378)	+ 1.5346 (0.0097)	+ 6.152 (0.1124)		
Moyale	+ 1.5914 (0.0041)	+ 5.0388 (0.1325)	+ 0.8997 (0.0022)		
Yabello	+ 0.1097 (5E-05)	− 2.8724 (0.0823)	+ 3.935 (0.1326)		

Moyale	Annual temperature	Genna	Adolessa	Hagaya	B.Hagaya
MAX TMP	+ 0.037 (0.0822)	0.0582 (0.2568)	+ 0.0581(0.4025)	− 0.0059 (0.0041)	0.228 (0.0764)
MIN TMP	− 0.0089 (0.0046)	− 0.0582 (0.0367)	+ 0.0288 (0.0466)	0.0152 (0.0155)	− 0.0975 (0.2029

^a^Slope and ^b^R^2^.

#### Nonparametric trend analysis

Trend analyses were conducted using MK and Sen’s slope estimators for seasonal and annual precipitation at each station. The summary statistics obtained for the main, short-season, and annual precipitation are presented in Table 4. Similarly, the MK trend analysis results for the maximum and minimum temperatures at the Moyale station, including the monthly trends, are presented in Tables 5, 6.

**Table 4:** Seasonal and annual precipitation MK test results at selected stations in the Borana zone. Source (Author): Computed based on data from the NMA of Ethiopia.

		Sen’s slope	Kendall’s tau	Mann Kendall (S)	Var (S′)	P value (two-tailed)	alpha	Test interpretation	Trend
ChewBet	Genna	− 3.8	0.05	4	196	0.83	0.05	Accept	NST(−)
	Hagaya	6.25	0.24	20	196	0.17	0.05	Accept	NST(+)
	Annual	0.001	0.02	7	2301	0.9	0.05	Accept	NST((+)
Finnchwha	Genna	− 12.8	− 0.06	− 6	276	0.76	0.05	Accept	NST(−)
	Hagaya	22.04	0.21	18	196	0.22	0.05	Accept	NST(+)
	Annual	0.003	0.06	28	3138	0.63	0.05	Accept	NST(+)
Moyale	Genna	− 4.7	0	0	374	1	0.05	Accept	NST(−)
	Hagaya	3.28	0.11	15	375	0.47	0.05	Accept	NST (+)
	Annual	− 0.005	− 0.08	− 33	3142	0.57	0.05	Accept	NST(−)
Yabello	Genna	− 11.84	− 0.22	− 24	276	0.17	0.05	Accept	NST(−)
	Hagaya	9.65	0.19	20	276	0.25	0.05	Accept	NST(+)
	Annual	− 0.001	− 0.03	− 15	3142	0.8	0.05	Accept	NST(−)

**Table 5:** Monthly, seasonal and annual maximum temperature MK test results for Moyale station.

Variable	Sen's slope	Mann Kendall (S)	Kendall's tau	Var (S)	p value (two-tailed)^a^	alpha	Test interpretation	Trend
Jan	0	82	0.17	3803	0.19	0.05	Accept	NST (+)
Feb	0	135	0.27	3802	0.03	0.05	Reject	ST (+)
March	0	157	0.32	3802	0.01	0.05	Reject	ST (+)
April	0	87	0.18	3802	0.16	0.05	Accept	NST (+)
May	0	144	0.29	3803	0.02	0.05	Reject	ST (+)
June	0	190	0.38	3803	0	0.05	Reject	ST (+)
July	0	186	0.38	3803	0	0.05	Reject	ST (+)
August	0	113	0.23	3802	0.07	0.05	Accept	NST (+)
September	0	106	0.21	3803	0.09	0.05	Accept	NST (+)
October	0	− 69	− 0.14	3802	0.27	0.05	Accept	NST (−)
November	0	− 183	− 0.37	3793	0	0.05	Reject	ST (−)
December	0	118	0.24	3794	0.06	0.05	Accept	NST (+)
Genna	0.056	27	0.2	375	0.18	0.05	Accept	NST (+)
Adolessa	0.171	65	0.48	375	0	0.05	Reject	ST (+)
Hagaya	− 0.053	− 13	− 0.1	375	0.54	0.05	Accept	NST (−)
BH	0.253	39	0.29	375	0.05	0.05	Reject	ST (+)
Annual mean TEM	0	76	0.15	3803	0.22	0.05	Accept	NST (+)

^a^An approximation has been used to compute the p value.

**Table 6:** Monthly, seasonal and annual minimum temperature MK test results for Moyale station.

Variable	Sen's slope	Mann Kendall (S)	Kendall's tau	Var (S)	P value (two-tailed)	Alpha	Test interpretation	Trend
Jan	0	− 31	− 0.06	3802	0.63	0.05	Accept	NST (−)
Feb	0	46	0.09	3803	0.47	0.05	Accept	NST (+)
Mar	0	46	0.09	3803	0.47	0.05	Accept	NST (+)
Apr	0	49	0.1	3802	0.44	0.05	Accept	NST (+)
May	0	2	0	3803	0.99	0.05	Accept	NST (+)
Jun	0	144	0.29	3803	0.02	0.05	Reject	ST ( +)
Jul	0	206	0.42	3803	0	0.05	Reject	ST (+)
Aug	0	185	0.37	3802	0	0.05	Reject	ST (+)
Sep	0	146	0.29	3803	0.02	0.05	Reject	ST (+)
Oct	0	212	0.43	3803	0	0.05	Reject	ST (+)
Nov	0	74	0.15	3803	0.24	0.05	Accept	NST (+)
Dec	0	45	0.09	3802	0.48	0.05	Accept	NST (+)
Genna	0.1148	21	0.18	309	0.26	0.05	Accept	NST (+)
Adolessa	0.131	66	0.56	315	0	0.05	Reject	ST (+)
Hagaya	0.136	49	0.42	309	0.01	0.05	Reject	ST (+)
BH	− 0.031	− 6	− 0.05	342	0.79	0.05	Accept	NST (−)
Annual mean TEM	0	13	0.03	3462	0.84	0.05	Accept	NST (+)

In the Mann–Kendall test results (S), a positive S value indicates an increasing trend, while a negative value indicates a downwards trend based on its significance. The significance test is based on rejecting or not rejecting the null hypothesis (Ho, i.e., no trend in the data series) at a 5% significance level (alpha value). If the p value is below the threshold of significance (p < 0.05), then the null hypothesis can be rejected, and the prevailing trend (increasing or decreasing) can be considered significant. Kendall’s tau is a nonparametric rank correlation coefficient that measures the strength of the relationship between the observed element (e.g., precipitation or temperature) and time. The higher the value of the coefficient is, the stronger the relationship is, and vice versa. The estimated Sen's slope further provides further information about the trend of the observed element. A positive Sen's slope reveals an upwards trend, while a negative Sen’s slope suggests a downwards trend.

### Characterizing drought and its severity

The seasonal rainfall anomaly index (ARAI) was calculated based on Eq. (1) presented above for each of the two main rainy seasons of the observed year at selected stations. The index values were further assessed against the 9-class scheme proposed by Van Rooy^36^, ranging from extremely wet (≥ 3.00), very wet (2.00 to 2.99), moderately wet (1.00 to 1.99), and slightly wet (0.50 to 0.99) to near normal (− 0.49 to 0.49), slightly dry (− 0.99 to − 0.50), moderately dry (− 1.99 to − 1.00), very dry (− 2.99 to − 2.00) and extremely dry (≤ − 3.00). Counts of wet and dry rainfall conditions corresponding to various severities for the two seasons are summarized in Table 7.

**Table 7:** Comparisons of seasonal RAI values at selected stations for the 1990–2017 period. Source (Author): Computed based on data from the NMA of Ethiopia.

Rainfall condition class	Chew Bet		Finchwuha		Moyale		Yabello	
	MAM	SON	MAM	SON	MAM	SON	MAM	SON
Extremely wet	0	1	0	1	0	2	0	0
Very wet	1	1	0	0	2	0	0	1
Moderately wet	3	3	3	4	1	2	2	2
Slightly wet	3	3	3	2	0	1	4	5
Near normal	7	7	10	7	13	5	11	9
Slightly dry	4	1	5	2	4	5	2	2
Moderately dry	5	1	5	3	5	3	7	1
Very dry	1	2	1	4	1	1	1	6
Extremely dry	4	9	0	4	2	9	0	1
Study period	28	28	27	27	28	28	27	27

The overall observed years at each station were classified into three categories, dry, normal, and wet years, based on the ARAI. In addition, the probability of occurrence of each category was calculated based on Eq. (2). The drought recurrence interval or return period was computed using Eq. (3). The result is presented in Table 8.

**Table 8:** Occurrence of dry seasons and dry-season intervals based on the RAI (1990–2017).

	Study period	Dry years		Normal		Wet years		Recurring interval of dry season	
		MOM	SON	MOM	SON	MOM	SON	MOM	SON
Chew Bet	28	14	13	7	7	7	8	2	2.2
	Probability	50%	46%	25%	25%	25%	29%		
Finchwha	27	11	13	10	7	6	7	2.5	2.1
	Probability	41%	48%	37%	26%	22%	26%		
Moyale	28	12	18	13	5	3	5	2.3	1.6
	Probability	43%	64%	46%	18%	11%	18%		
Yabello	27	10	10	11	9	6	8	2.7	2.7
	Probability	37%	37%	41%	33%	22%	30%		

### Normalized Difference Vegetation Index (NDVI) analysis

#### Parametric trend analysis

**Figure 4:**
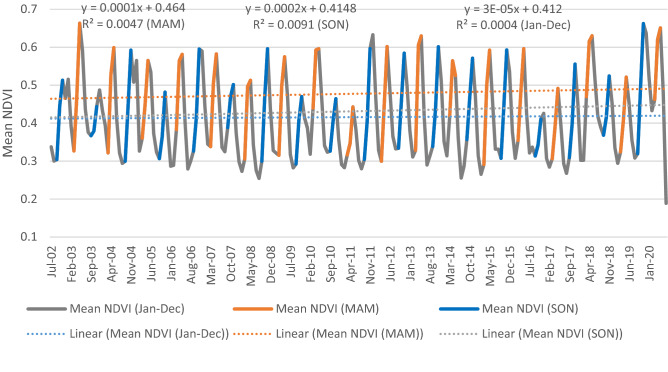
Trends of the yearly and seasonal-mean NDVI from 2002 to 2020 in the Borana zone. [Source (Author): computed based on data from FEWS NET].

The calculated monthly mean NDVI from 2002 to 2020 was plotted in a line graph over time to ascertain any observable trends, and the results obtained for the annual period and the two main seasons are presented in Fig. 4. A simple linear regression equation was generated to predict the monthly NDVI for a unit change of the dependent variable (time in a year). In addition, linear trend lines and R-squared statistics were generated from the built-in tool to determine the direction of change and accuracy of the fit. This information corresponding to the annual period and two seasons is incorporated in the line graphs.

The nonparametric trend analysis method incorporating the M–K test and Sen’s slope was used here to determine the direction, statistical significance and magnitude of the trends of the Dekadal NDVI, and the results are presented in Table 9.

**Table 9:** M–K test of NDVI anomalies from 2002 to 2020 in the Borena zone.

	NDVI anomaly (yearly)	NDVI anomaly (MAM)	NDVI anomaly (SON)
Mann Kendall (S)	3121	228	1502
Var (S')	30,583,987	467,964	467,961
Kendall's tau	0.015	0.018	0.117
Sen's slope	− 0.0012	0.0144	− 0.0281
P value (two-tailed)	0.573	0.74	0.028
alpha	0.05	0.05	0.05
Test interpretation	Accept	Accept	Reject
Trend	NST (−)	NST (+)	ST decreasing

### Local perspectives on climate variability, climate change, and drought

**Figure 5:**
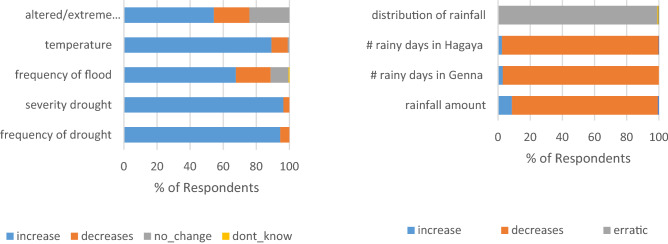
Respondent perceptions on climate variabilities and change parameters.

Local knowledge is vital for understanding past and present patterns of climate change and related hazards, particularly in contexts with weak weather and climate monitoring systems. Household perceptions of climate variabilities and changing parameter responses were assessed herein using the past 10 years as a reference. The analysis and summary are presented in Fig. 5. The youngest to oldest age groups (20 to 95 years old) were interviewed to help bridge views and opinions across generational gaps. Most of the respondents (78%) had resided in the area for an extended period (20 years was a modal value) in the same location, which helped us reasonably assume their familiarity with observable changes in their area. Only 12% of the respondents claimed to have migrated from other areas in the last 10 years or less.

Households clearly indicated that the frequency and severity of drought and temperature have increased compared to the last 10 years. Households were also undivided in their responses regarding the reduction of the rainfall amount and number of days in the two main seasons. They also unanimously agreed on the erratic nature of the rainfall pattern in recent time compared to over the past 10 years. The frequency of flood events and extreme temperatures were overall perceived to have increased, although the response of the households were not as united as those corresponding to the previous indicators.

The KII assessment provided further insight into climate change parameters in addition to affirming the household analysis results presented in Fig. 5. The informants widely cited rainfall pattern changes as evidence of their observed change. The informants compared rainfall patterns in terms of their timing, amounts, and distributions in the present day and over the past 30 years to substantiate their claims, as presented in Table 10.

**Table 10:** Comparisons of past and current climate variabilities and change parameters. Source: Key Informants Interview, July–August 2019.

Rainfall pattern	Main rainy season (Genna)		Short rainy season (Hagaya)	
	Past	Current	Past	Current
Onset of rains	February	March/April	August	October
Cessation of rains	April	End of April	October	October
Months/days in the rainy season	3 (180 days)	1–2 (30–60 days)	3	Unreliable (a month or less
Probability of missed/failed season	Unlikely to occur	May or may not occur	Likely to occur	Likely to occur (increased)
Rainfall amount	Adequate	Decrease/inadequate	Adequate or inadequate	No rain/rain rarely received
Early shower	January	No shower as per recent trend	July	No shower at all recently
Days of long dry season	90	100–140		
Timing of rainfall	Predictable	Unpredictable	Predictable	Extremely unpredictable
Distribution	Well-distributed	Erratic	Erratic	Very erratic
	Past		Current	
Warming temperatures	Dry season		Increase in temperature observed regardless of the season or day/night	
Drought occurrence	Once every 7–8 years		Recurrent (frequency increased)	
Drought severity			Severity increased	

The informants were unanimous in terms of the rising temperature trend in the area and linked this trend with the changing pattern of rainfall. In the past, the weather during the long dry season was often characterized by relatively high temperatures during the day and low temperatures, including an uncomfortable coldness, at night. However, low temperatures (coldness) are observed during both day and night in the cold dry season. In recent times, however, increasing temperatures have been observed regardless of the season or day/night.

Drought recurrence and severity were other parameters the informants compared to support their assertion of climate change. For example, Malbee grazing managers asserted that as droughts became more frequent, even before livestock keepers recovered from one drought, another drought hit the area. The historical timeline collated from the key informant interviews provided further insights into the temporal dimensions of major hazards. The synthesis of major drought events and associated disasters the population has experienced since the early 1960s and their chronological characteristics corresponding to the Gadaa calendar are presented in Annex 5. This synthesis can help us understand the causes and effects of the events and put the research into a historical perspective.

Drought and dry spells are among the major hazards threatening the livelihoods of the population group. The sample household ranking order of these common hazards further augments key informant assessments. Erratic rainfall amounts and distributions, including drought events, ranked first among the common hazards faced by sample households. Our analysis of extracted digitized data recorded from 1994 to 2013 revealed that drought was the most common disaster during this period. Drought events were widely distributed spatially across the studied zone, while other hazards affected localized areas of the zone, as presented in Annex 6.

#### Causes and aggravating factors of climate change

Most of the informants acknowledge climate variability and drought as part of the Borana ecosystem. Informants suspect that observable change in climate started to manifest either during Gadaa Boru Guyo (1986–1993) or as early as in Jeledesa Liben (1962–1968). However, the worsening of these changes by each Gadaa, regardless of when it started, was the overwhelming consensus of the informants.

Informants were quite divided as to the causes and contributing factors of climate change. The majority of the elders, however, strongly believed that climate change is an “act of God” and did not subscribe to the scientific basis of human-interference (anthropogenic) causes of climate change. Some of the informants attributed the changes to the weakening of traditional customs and practices.

One of the grazing managers and most of the KIs from the government acknowledged the link between deforestation and climate change. The KIs highlighted the gradual deforestation pocket of the forested area in Borana (Miyo, Arero, and Yabello) due to various human activities and believed that these changes contributed to local climate change, particularly to the warming of temperatures. Land use and land cover change (LULC) observed in the area due to internal and external factors might also have contributed to or aggravated local climate change.

The informants from government institutions did not discount the aspect of the global climate change process, i.e., greenhouse gas (GHG) emissions aggravating the local climate conditions. Methane gas (CH4) emitted through livestock disposal has its own share of concern given that livestock-keeping is the predominant means of maintaining livelihood in the area.

## Discussion

High rainfall variabilities observed in the study area are similar to the patterns recorded in Eastern African drylands, which have very high inter-annual variability in the length of the growing season, particularly for areas with bimodal distributions^37^. East Africa experiences some of the largest inter-annual rainfall variations in the world, and there are many different drivers of this variability^38^. However, the higher temporal and spatial rainfall coefficients of variation in the study area compared to the estimate (15–25%) for Eastern Africa^37^ are concerning and indicative of the increasing extreme rainfall events.

No statistically significant trends were observed in the seasonal, or annual mean rainfall at the four analysed stations. However, the observed downwards trend of the main-rainy-season precipitation among the four stations mirrored the widely held perception of the community. The upwards trend of the short rain season observed, although not statistically significant, was quite contrary to the household responses and qualitative and NDVI trend analyses. For example, the vegetation condition trend (NDVI) of the short rainy season showed a statistically significant downwards trend that was strongly related to the rainfall received in semiarid regions of Africa^39^. Qualitative and household analyses further conclusively suggested that the amount of rainfall decreased and became insufficient, notably for the short rainy season, compared to the past. Some studies in Ethiopia have identified downwards trends in precipitation in some parts of the country, mainly in the eastern, southern, and south-eastern regions^40,41^. The key informant comparisons of past and current rainfall pattern timing, amounts, and distributions presented in Table 10 provide substantial and contextual information as to unfavourable precipitation, temperature, and drought trends. Quantifying observed trends in precipitation is more challenging than quantifying those in temperature due to the lack of reliable data and variability on multiple temporal and spatial scales. However, significant declines in the Long Rains from March to May over the Horn of Africa were observed in the 1980s and 1990s^42^, followed by a recent recovery^43^.

The trend analyses of the mean monthly, seasonal, and annual maximum and minimum temperatures at Moyale station showed increasing (warming) trends overall, although the results were mixed in terms of their statistical significance. The February, March, May, June, and July monthly maximum mean temperatures were statistically significant. Similarly, the minimum mean temperature for the June-October period exhibited positive and statistically significant trends. Seasonally, statistically significant warming of maximum temperatures was observed in the cold and long dry seasons, while a similar pattern was observed in the cold dry season and short rainy season for the minimum temperature at the 5% level of significance. The key informants were highly unanimous about the warming of temperatures in the study area regardless of the season or time of day/night in recent times. Households also clearly indicated that temperatures, including extreme temperatures, have increased in the area compared to the past 10 years. Early analyses^44^ suggested that the temperature increases in the semiarid lowland regions of Ethiopia were more pronounced than the national average changes as well as those in highland regions in the country. These regions, particularly Borana, Guji, and South Omo, experienced temperature increases of 0.40 °C per decade from 1950 to 2000, further supporting the findings of this study.

The pattern observed in this study is in conformity with the two broader consensuses and evidence of climate change in Eastern Africa^45^. Increasing temperature trends from climate baselines are among the broad pieces of evidence obtained at the regional level. Second, the overall rainfall trend assessments were weak and hard to detect despite either increasing or increasing trends observed depending on the specific locations and seasons.

The observed high rainfall variability entails a high degree of rainy-season uncertainty in the study area in general, particularly for the short rainy season. This finding implies that the study area, particularly the low-land area, is exposed to a relatively high degree of climate risk, particularly due to extreme precipitation, because of the relatively high inter-annual and seasonal rainfall variabilities and relatively low precipitation. Our seasonal rainfall anomaly analysis suggests that the study area faced probabilities of experiencing dry conditions at different intensities (− 0.5 and less) during the main and short rainy seasons of 35–50% and 35–65%, respectively. This probability implies that 4–5 and 4–6 dry events of different intensities occurred every 10 years during the main and short rainy seasons, respectively. The stations located in lowlands had higher dry-year frequencies (including higher intensities) than the stations in mid/higher elevations, linked with their exposure to a relatively low amount of seasonal rainfall and high variabilities. On average, droughts of different intensity degrees occur every 2 to 3 years in consecutive or non-consecutive seasons in the study area, as the recurrence interval or return period calculation results of the observed data suggest. The observed temperature increases will aggravate the prevailing dry conditions by enhancing evaporation, thereby reducing surface water and drying out the soils and vegetation. Rainfall and evapotranspiration are two major climatic factors affecting agricultural production^46^, and agricultural water resources face two major problems. Climate extremes significantly impact livestock productivity in eastern and western Africa. The increasing frequency and intensity of droughts, changes in water availability, and increasing patterns of temperature and rainfall variability all profoundly threaten the livelihoods of people living in drought-prone areas and the existence of society in arid and semiarid remote regions^47,48^.

The empirical results reported herein should be considered in light of some limitations. The climate risk analysis presented in this paper was based on limited observed rainfall and temperature data due to the unavailability of high-quality data from meteorological stations in the area of interest. In this research, we performed the necessary quality and homogeneity tests to ensure the integrity of the results. The use of gridded data that blend station-observed data and satellite observations or reanalysis data is recommended in a future study to broaden the scope and application of the findings of this research.

## Conclusion and recommendation

Recently, unfavourable drought, climate variability, and climate change trends have been observed in the study area, according to most households and key informants. Climate data provided further credence to these household and community perceptions regarding warming temperature trends, increasing rainfall variability, and recurring dry and drought conditions. However, the climate data results did not show any unified pattern regarding the local characterization due to the limited availability and quality of long-time-series climate data in the study area. These findings highlight the need to integrate local and indigenous knowledge in climate risk studies in addition to measured climate data.

There was no consensus among the stakeholders on the causes and aggravating factors of the observed changes, though these factors have implications for adaptation actions. The authors recommend the importance of participatory workshops on climate change and its impact on the Borana pastoral system by bringing actors and the affected populations together and forging paths forward. Such workshops will create a common understanding of the problem (framing the problem) related to public policy. The workshops will further provide opportunities to create platforms hitherto absent in the area that can regularly meet to discuss the issue of climate change. The analysis and synthesis of the observed climate and qualitative data highlighted in this study serve as valuable input for building consensus.

Drought and dry spells were the major hazards cyclically grappled with by the pastoralists and agro-pastoralists in the study area. Drought events are widely distributed spatially across the studied zone compared to other hazards. A historical timeline collated from the key informants and focus group discussions provided further insights into the temporal dimensions of major hazards. The analysis captured major drought events the population has experienced since the early 1960s and their characteristics. The major drought events clearly demonstrate the “downwards spiral” of the livelihoods of the population. The RAI analysis suggested that drought events with different intensity degrees are recurring in 2- to 4-year intervals in the study area^18^, reiterating concern for the vulnerability of pastoral populations in the arid and semiarid zones of East Africa to recurring drought events despite the wide range of socioeconomic and cultural benefits from livestock rearing.

This research used drought indices that quantify climatic anomalies from observational and remotely sensed data to characterize the drought severity, duration and frequency; to date, such an analysis has not been performed. One cited study^49^ found indices to be a very useful tool in communicating complex climatic functions, including drought severity, in an easily understandable way to a wider audience. This study demonstrates the added value of such tools in decision-making in the study area. This kind of information communication tool is urgently required as the frequency, intensity, and cost of natural hazards are expected to further increase in the coming decade, as indicated by the IPCC in its fifth assessment report^50^.

## Supplementary Information

Supplementary Information 1.

Supplementary Information 2.

Supplementary Information 3.

## Data Availability

The meteorological data that support the findings of the climate aspect of this study are available from the National Meteorology Agency of Ethiopia, but restrictions apply to the availability of these data, which were used under licence for the current study and are not publicly available. Data are, however, available from the authors upon reasonable request and with permission of the Agency. Furthermore, household survey datasets and supplementary information (annexes) are available from the corresponding author upon reasonable request.
