# Supplementary material for: Understanding physical climate risks and their implication for community adaptation in the borana zone of southern Ethiopia using mixed-methods research
Source: Sci Rep. 2023 Apr 27;13:6916. doi: 10.1038/s41598-023-34005-1 (PMC10140045; doi:10.1038/s41598-023-34005-1)
Supplement: Supplementary file 1 — Supplementary Information 1. [file 41598_2023_34005_MOESM1_ESM.docx]

**ANNEXES**

**Annex 1: Sampling procedures, sample size, and its distribution in the study area**

Pastoral and agro-pastoral in the zone are considered the population group of the research. This group was dispersed over an expansive area in the zone, thus further subdivided into woreda (districts), pastoral associations (PAs), and villages (ollas) administratively. Considering the variability and size of the zone, the research employed a two-stage cluster sampling method. The primary sampling unity (PSU)-the clusters were selected at the first stage and followed by the households, which is considered secondary sampling unity (SSU).

The sample size (n) of the study was determined based on a two-stage cluster sampling, levels of acceptable error, level of confidence, design effect, and non-response rate using the below equation.

$n=\frac{{d*z}^{2}*p*q}{a^{2}}+nr*\frac{{d*z}^{2}*p*q}{a^{2}}$ Equation 1: Sample size determination

| **n** | Sample size |
| --- | --- |
| **z** | alpha risk expressed in Z-score (this is 1.96 for 95% confidence level) |
| **p** | Expected prevalence (unknown assumed to be 50%) |
| **q** | 1-p (50%) |
| **d** | design effect (assumed to be 2 although this is on the high side and it can be reduced to 1.5 |
| **a** | Absolute precision (margin of error ±5%) |
| **nr** | Non-response rate (assumed to be 10%) |

$$n=\frac{2*{1.96}^{2}*0.5*0.5}{{0.05}^{2}}+(10\%*768)=845 HHs$$

The List of 162 rural PA collated from thirteen districts of the zone used as PSU. Forty-five sample clusters were selected based on 845 households' sample sizes and Proportional Probability Sampling (PPS). However, Moyale and Guchi woreda (adjacent to each other) which constituted 13 of the 45 sample clusters were excluded from the final sample clusters due to the prevailing conflict. The sample plan was reduced to 627 households and 32 clusters.  In addition, three clusters were not covered due to insecurity which contributed to a further reduction in the number of households to be interviewed. Finally, 529 households out of 627 planned (84% response rate) were interviewed in 11 of 13 districts of the Zone (refer Annex 1 for map of sample household distribution). The engagement of the sample household’s in cultural rituals, vaccination, meetings, and other personal matters affected the level of the response despite proactive planning from the research team.


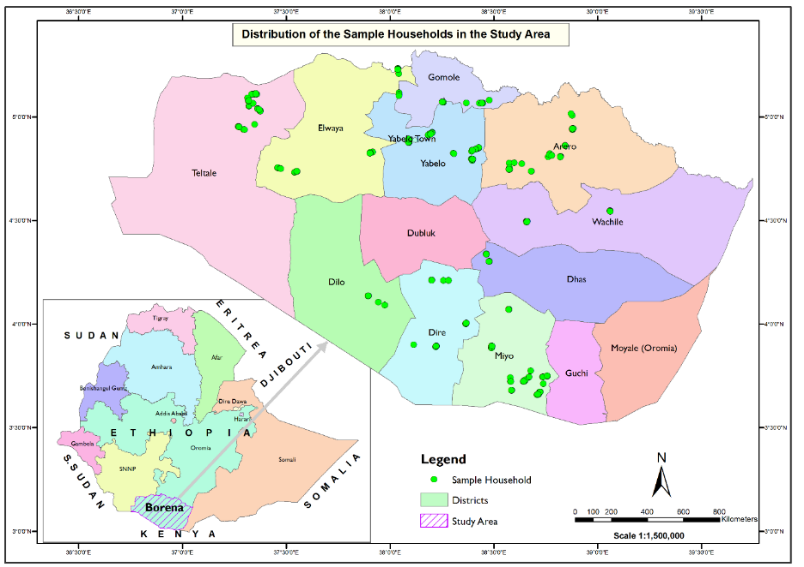


Source: HDX-Ethiopia Shape file and GPS points of Household survey

Annex 2: Distribution of the sample clusters FGDs facilitated in the study area

 

Source: HDX-Ethiopia Shape file and GPS points of FGDs collated

*Annex 3:* M-K Test seasonal and annual MAX temperature of Moyale station from 1986-2017

|  | Genna | Adolessa | Hagaya | BH | Annual Mean TEM |
| --- | --- | --- | --- | --- | --- |
| Mann Kendall (S) | 27 | 65 | -13 | 39 | 76 |
| Var (S) | 375 | 375 | 375 | 375 | 3803 |
| Sen's slope | 0.056 | 0.171 | -0.053 | 0.253 | 0 |
| Kendall's tau | 0.2 | 0.48 | -0.1 | 0.29 | 0.15 |
| p-value (Two-tailed) | 0.18 | 0 | 0.54 | 0.05 | 0.22 |
| alpha | 0.05 | 0.05 | 0.05 | 0.05 | 0.05 |
| Test Interpretation | Accept | Reject | Accept | Reject | Accept |
| Trend |  | ST* increasing |  | ST increasing |  |

*An approximation used to compute the p-value. ST*: significant Trend

*Annex 4:* M-K Test seasonal and annual MIN temperature of Moyale station from 1986-2017

|  | Genna | Adolessa | Hagaya | BH | Annual Mean TEM |
| --- | --- | --- | --- | --- | --- |
| Mann Kendall (S) | 21 | 66 | 49 | -6 | 13 |
| Var(S) | 309 | 315 | 309 | 342 | 3462 |
| Kendall's tau | 0.18 | 0.56 | 0.42 | -0.05 | 0.03 |
| Sen's slope | 0.1148 | 0.131 | 0.136 | -0.031 | 0 |
| P-value (Two-tailed) | 0.26 | 0 | 0.01 | 0.79 | 0.84 |
| alpha | 0.05 | 0.05 | 0.05 | 0.05 | 0.05 |
| Test Interpretation | Accept | Reject | Reject | Accept | Accept |
| Trend |  | ST increasing | ST increasing |  |  |

Source (Author): Computed based on data from NMA of Ethiopia

| Annex 5: Chronology of major drought events and its impacts in Borana by Gadaa   \| Reigns of Abbaa Gadaa* \| Abba Gadaa \| Key characteristics/ impact / coping \| \| --- \| --- \| --- \| \| 1962-1968 \| Jeledesa Liben Guyo \| No major drought. Conflict locally called “Olki Roba” characterized his Gadaa. \| \| 1970-1977 \| Goba Bule Dabasa \| Characterized by drought and conflict. Borana experienced two droughts during his Gadaa. The severe one was locally referred to as “Ola aduni Dote”. Livestock mortalities were significant and the human population was affected by diseases related to starvation such as bloody dysentery and Edema (“bobos”) that contributed to human fatalities.    Droughts prior to this Gadaa were manageable with exception of the worst droughts in the history of Borana called “Ola*Kolege”.*The impacts started to worsen from his Gadaa. Relief food and livestock feed (molasses and dry feed) distribution started that saved human lives and livestock from excess mortalities \| \| 1978-1985 \| Jilo Aga Adi \| Characterized by drought and conflict. Livestock mortalities were very high; the remaining one was very weak and was not able to fetch any price on the market. The impact was aggravated by the total unavailability of grain on the market because of the extensive drought in the country. This aggravated food access to drought-stricken populations and contributed to high human fatalities as a result of hunger and malnutrition  Most of the affected people distressfully migrated and joined relief camps. The relief camps such as Dolo Makala, Miyo, etc. were established during this period \| \| 1986-1993 \| Boru Guyo Boru \| Drought, conflict, and diseases (livestock/human) characterized this Gadaa. The drought named “drought of black flies” was related to swarms of *black flies covering* the corpses of livestock*. C*onflict compounded widespread drought. The inter-ethnic conflict and displacement were widespread, and the migration of livestock aggravated the situation. The change of government that happened towards the end of this Gada, contributed to the situation.    Livestock diseases Babesiosis (locally called “Birte”) and Foot-and-Mouth (FMD-Oyali) contributed to livestock mortalities more than the drought.    Starvation was widespread and human diseases contributed to the suffering of the affected population. The human population have limited access to livestock product access and was affected by hunger and malnutrition-related diseases (“Lakama”) and fatalities reported \| \| 1994-2001 \| Boru Madha Galma \| Borana has encountered two droughts in his Gadaa i.e. fourth year and the last year of his Gadaa.  The latter one was considered a major. This drought named “windy drought” or locally called “Ola Bube” related to very windy conditions prevailed. First, the Genna season was unfavorable and subsequently missed Hagaya totally. The dry season extended and reached about 140 days before receiving the rain. High livestock mortalities and some livestock keepers “abnormally” migrated to Burji, Konso, and Sagan River to save their livestock. Livestock-keepers who remained in Borana territory lost their livestock completely. \| \| 2002-2009 \| Liban Jeledesa Liban \| No major drought was reported except dry spell. The impact on livestock was limited. An extended period of his Gadaa (six years) claimed to be better off and only two years were considered as unfavorable with the dry spell \| \| 2010-2017 \| Guyo Goba Bule \| The Borana experienced two droughts during his Gadaa when he was taking the power and last year of his term. The later drought was considered as severe in its impact. Borana lost significant livestock (descended to impoverishment) as some of the elders characterized it    The drought in Guyo Goba was considered one of the worst droughts in Borana in recent history. Elders further ranked the drought in terms of impact as follows (Guyo Goba, Boru Madha, Boru Guyo,) \| \| 2018-2025 \| Kura Jarasso Kura \| Two years of his term. No major drought except localized dry spells. Residual impacts of previous droughts widely persist. Conflict remains a challenge for the livelihoods of  the population \|   Source: Key Informants Interview and Focus Group Discussion, July-August 2019  * The Abbaa Gadaa serves serve for a single term of eight years. |
| --- | --- | --- | --- | --- | --- | --- | --- | --- | --- | --- | --- | --- | --- | --- | --- | --- | --- | --- | --- | --- | --- | --- | --- | --- | --- | --- | --- |

Annex 6: Number of reported disasters in Borana zone from 1994-2013

| **Disaster Event** | Arero | Dire | Miyu | Moyale | Teletele | Yabelo | Total | % of the total reported event |
| --- | --- | --- | --- | --- | --- | --- | --- | --- |
| Drought | 16 | 29 | 9 | 19 | 18 | 17 | 108 | 60% |
| Biological | 2 |  | 8 | 3 | 2 | 5 | 20 | 11% |
| Conflict | 1 |  |  | 17 |  | 1 | 19 | 10% |
| Plague |  | 4 |  | 4 | 3 | 2 | 13 | 7% |
| Flood | 1 |  | 4 | 3 | 2 | 2 | 12 | 7% |
| Fire/Forest Fire | 5 |  |  |  |  | 2 | 7 | 4% |
| Landslide | 2 |  |  |  |  |  | 2 | 1% |
| Total | 27 | 33 | 21 | 46 | 25 | 29 | 181 |  |

Source: UNDRR, 2016
